# How the presence of others shapes the user experience of service robots

**DOI:** 10.3389/frobt.2025.1538711

**Published:** 2025-04-16

**Authors:** Stefan Tretter, Pia von Terzi, Sarah Diefenbach

**Affiliations:** Department of Psychology, Ludwig-Maximilians-Universität München, Munich, Germany

**Keywords:** service robot, HRI, observer, public space, expressivity, psychological needs, user experience

## Abstract

In the age of mobile and self-service technologies, human-computer interaction (HCI) often takes place in public settings. Such interactions can be considered a performance in front of others, when the relationship with potential observers may affect user preferences for different interaction styles. From a psychological perspective, public interactions may feel embarrassing or disturbing, but they also provide the opportunity for favorable self-presentation or connection with others. The present study investigated how the presence of different observers (i.e., acquaintance, stranger) emphasizes different psychological needs and, in turn, affects preferences for more or less expressive interactions with a service robot. Results show that users’ need for relatedness was higher when imagining a robot interaction with close observers, while popularity was more important with unknown observers. Relatedness was directly linked to a preference for more expressive interactions, regardless of the expected outcome. In contrast, popularity led to stronger expressivity preferences only when users anticipated a successful interaction for which they could take credit. Our research provides valuable insights into the impact of user-observer-relationship on public HCI, and can inspire designers to take into account how present others and users’ expectation of successful outcomes may call for different degrees of expressivity in interaction design.

## 1 Introduction

A major theme of modern life is our increasing reliance on technologies. In addition to the myriads of personal devices that shape our private lives, we also encounter more and more innovative and unfamiliar technologies in public spaces. Companies are already using ticket machines, ordering terminals, or self-checkouts to replace human staff and empower the customer, while the service robot market is predicted to grow by a multiple in the next few years (Tojib et al., 2022).

Accordingly, users increasingly interact with more or less unfamiliar devices while other people are around, potentially witnessing, judging, or even joining them. Therefore, those interactions become akin to public performances. Such performances may evoke feelings of embarrassment or pride, depending on whether we struggle or succeed, but they may also create a sense of shared experiences. Those feelings are grounded in the basic psychological needs for popularity and relatedness, two inherently social needs, whose fulfillment during product interactions supports positive and meaningful user experiences (Hassenzahl et al., 2010).

Research addressing human-computer interaction in public space or context often treats present others as a potential risk or problem as they can disturb or be disturbed by the user interaction. Social acceptability often dictates the design of public technologies (Koelle et al., 2020), resulting in subtle or covert interaction patterns. However, we propose that the presence of others can also be a source of positive experiences in public, if they provide the opportunity to fulfill needs that require other people, like popularity or relatedness. But for these needs to be fulfilled, others must witness our interactions, which is why their expressivity, i.e., how extensive and noticeable they are, becomes paramount. In other words, interaction expressivity refers to how perceptible a user’s interaction is to an audience, influencing social dynamics. High expressivity, such as a loud, animated conversation, attracts attention, while low expressivity, like a quiet, private discussion, keeps the interaction more contained.

At the same time, when our interactions are more expressive, and thus may be witnessed by others, how we relate to those others becomes increasingly important. We suppose that individuals place greater importance on gaining acceptance and recognition (need for popularity) with strangers, i.e., observers they do not know, while prioritizing building and maintaining relationships (need for relatedness) with acquaintances, i.e., people who are emotionally closer to them.

To explore the impact of observer relationships on user experience, we conducted a vignette study that examined interactions with a service robot in a café setting. Service robots may be considered a subtype of self-service technologies, as they are “technological interfaces that enable customers to produce a service independent of direct service employee involvement” (Meuter et al., 2000, p. 50). However, compared to traditional self-service technologies such as check-out terminals or ticket machines, robots can engage with consumers on a social level (van Doorn et al., 2017; Mende et al., 2019) and are becoming increasingly common in public places like restaurants (Pieska et al., 2013).

In our study, we asked participants to indicate what would be crucial for an ideal interaction with a service robot in a public scenario when being observed by a person either close or unknown to them. Preferences for the fulfillment of different psychological needs (i.e., relatedness and popularity) and expressivity (i.e., how extensive, obvious, and/or noticeable the interaction should be) were assessed, as well as people’s cognitive appraisals of the expected encounter (i.e., success expectation and its attribution). We then take these insights and discuss implications for theoretical research and practical design, presenting service robots not just as functional tools but as facilitators of meaningful and context-sensitive user experiences. Finally, we address the study’s limitations and propose directions for future research.

## 2 Related work and research gaps

Understanding how social context shapes interaction preferences with service robots is crucial for designing technologies that align with user expectations and thus foster acceptance. While extensive research has examined the factors influencing the dyadic interaction of humans with robots, the impact of adding different observers to these situations and how they may affect users’ needs and preferred interaction styles remains underexplored.

### 2.1 Observers' effect on technology interactions

Self-service technologies (SSTs), including service robots, are increasingly deployed in public environments, where users interact under varying social influences. While studies have investigated individual acceptance factors such as ease of use, prior experience, and perceived risk, research on contextual influences remains limited. Meta-analytic evidence suggests that public settings significantly alter acceptance patterns, with technological anxiety intensifying in crowded or time-constrained environments (Gelbrich and Sattler, 2014; Blut et al., 2016). These findings highlight the need to examine observer effects in public technology interactions.

Insights from social psychology suggest that passive observers shape behavior through the “mere presence effect” (Guerin, 1986), where individuals modify their actions based on being watched. Similarly, social facilitation theory (Zajonc, 1965; Aiello and Douthitt, 2001) suggest that observer effects exist and that they depend on task difficulty. More recent research in consumer psychology also demonstrates that even non-participating social entities can influence service experiences, modifying user preferences and decision-making processes (Argo et al., 2005; He et al., 2012; Argo, 2020).

Despite growing interest in service robot acceptance, research on the impact of social environments on the interaction with service robots is scarce. Some human-robot-interaction (HRI) studies suggest that the presence of observers influences user perceptions of robots, yet comparative analyses across different observer conditions are largely absent (Holthöwer and van Doorn, 2022). But initial evidence indicates that psychological motivations influence user preferences for human versus robotic service staff, depending on social comfort levels (Tojib et al., 2022). Additionally, observers seem to influence perceived trustworthiness and emotional responses in user-robot interactions (Delgosha and Hajiheydari, 2021).

Psychological research also suggests that people’s motivations and experiences will significantly vary not only depending on whether an observer is present, but also on how close they feel to those observers (Boothby et al., 2016; Dubois et al., 2016). But while the presence of more or less psychologically close others (e.g., friends vs. strangers) has already been shown to influence regular service encounters (He et al., 2012; Qiu et al., 2018), its influence on service HRI remains yet unexplored.

### 2.2 Psychological user needs and expressivity in public interactions

Another potentially crucial but underexplored factor in HRI, as soon as it happens in crowded places, is interaction expressivity—the extent to which a user’s interaction with a robot is visible and noticeable to surrounding observers (Bruns et al., 2021). For example, voice-command interactions tend to be highly expressive because they involve audible communication that others can easily hear and interpret, often attracting attention. On the other hand, touchscreen interactions are relatively private, as they occur through tactile input that is less perceptible to those nearby, thereby reducing the social visibility of the interaction. Interaction expressivity is distinct from robot expressivity, which focuses on the robot’s ability to convey social and emotional cues, whether subtle or explicit. More specifically, it can be defined as “the ability for a robot to successfully communicate dynamic emotional states and intent in a social context during human-robot interactions through embodied communication” (Gomez et al., 2020, p. 1970). When talking about expressivity, we refer to interaction expressivity, i.e., how publicly observable the user-robot interaction is.

Prior research suggests that users adjust their behavior based on the observability of their interactions (Koelle et al., 2020). Expressive interactions can be socially rewarding when users seek validation or shared experiences (Desmet and Fokkinga, 2020). However, they may also trigger self-presentation concerns, particularly in unfamiliar social contexts (Goffman, 1959; Leary, 2019). For instance, while a user may comfortably place an order via voice with a waiter robot in a social setting, they might prefer a less conspicuous touchscreen interface in a pharmacy with strangers nearby. Despite these dynamics, little research has examined how observer presence influences user preferences for expressive interactions with service robots.

As humans, we have an innate human desire for a positive self-image (in terms of approved social attributes) that we want others to share (Goffman, 1955). This fundamental human need for social approval may well extend to technology interactions, where users engage in impression management—adjusting behaviors based on social visibility and evaluation in hopes of positive self-presentation (Leary, 2019). Public technology interactions introduce self-presentation risks, influencing whether users engage with or avoid technology in front of others (Koelle et al., 2020). However, prior research primarily emphasizes negative effects such as embarrassment and discomfort. Traditional perspectives on social acceptability in technology interactions focus on minimizing visibility to reduce negative judgments (Koelle et al., 2020). This approach overlooks potential benefits, such as enhanced social validation, status signaling, or collective experiences (Hassenzahl et al., 2010; Desmet and Fokkinga, 2020).

User experience (UX) research suggests that technological interactions fulfill fundamental psychological needs, including relatedness and popularity (Hassenzahl et al., 2010; Desmet and Fokkinga, 2020). Relatedness, i.e., the desire for social connection and shared experiences, and popularity, i.e., the motivation to gain recognition and validation, have both been shown to be an integral part of various positive technology interactions in public (von Terzi et al., 2021).

However, both needs are inherently different. When being observed, sharing an experience to foster relatedness becomes easier as interactions become more expressive and thus more noticeable. But a feeling of popularity is more contingent. Of course, others witnessing the interaction is still a requisite for gaining recognition. But we also have to succeed at it and be responsible for our success.

According to the control-value theory of achievement emotions (Pekrun, 2000; Pekrun, 2006) anticipated emotions and behavioral choices depend on perceived control and subjective value, i.e., is the task at hand important to us and do we feel able to successfully manage it (Pekrun et al., 2007). Applied to the feeling of popularity in public HRI, this suggests that a user’s preference for more expressive interactions is dependent on their expectation to successfully interact with the robot and whether they can take credit for this success (or blame for failure). This aligns with recent findings highlighting the role of performance expectations (Fan et al., 2020; Tojib et al., 2022) and blame attributions (Belanche et al., 2020a; Fan et al., 2020) in service robot interactions.

Current research in robot design has increasingly recognized the importance of affective and contextual factors, expanding beyond traditional models such as the Technology Acceptance Model (TAM; Davis, 1989). While service robots are generally expected to perform well on pragmatic measures by default, recent studies have begun to highlight hedonic factors as essential drivers for service robot adoption and use, particularly in public spaces (Lu et al., 2019; Alotaibi et al., 2022; Molinillo et al., 2022). This study contributes to this evolving research agenda by examining how observer presence shapes users’ needs for relatedness and popularity, and in turn expressivity preferences, offering new insights into the reason behind positive experiences of HRI in public spaces.

## 3 Hypotheses

Public settings are defined by the presence of others, which shapes individuals’ behaviors and interactions (Goffman, 1959). The presence of others can fulfill social needs by providing social interaction, e.g., support or validation. According to Deci and Ryan (2000), social interactions are key to fulfilling basic psychological needs like relatedness, which in turn can enhance motivation and satisfaction. The fulfillment of psychological user needs is a central aspect of good user experience, which is closely linked to the acceptance of technologies like service robots. For example, Hassenzahl (2010) emphasizes that designing user experiences is grounded in meeting users’ needs, leading to higher satisfaction and positive experiences, and Hornbæk and Hertzum (2017) discuss the strong connection between user experience and technology acceptance. Specifically, in the context of HRI, current research underscores the importance of placing psychological needs at the core of design processes (Janssen and Schadenberg, 2024) and the relevance of environment-related factors, such as public vs. private settings (Honig et al., 2018).

Given the outlined research gaps above regarding social context and experiential qualities, we set out to investigate the public interaction with service robots in a study that integrated the three basic pillars of service robot implementation (Belanche et al., 2020b): service encounter characteristics (here: the relationship to observers), customer features (here: the users’ dominant psychological needs), and robot design (here: the degree of expressivity of an interaction). Based on the reviewed literature we scrutinized the following assumptions.

### 3.1 Observer relationship and psychological user needs

The presence of close vs. unknown observers influences social motivations in public interactions. When interacting in front of close others, individuals prioritize relatedness, seeking shared experiences and social connection. In contrast, when observed by strangers, individuals are more likely to focus on popularity, aiming to enhance status or manage impressions. Thus, we expect that users interacting with a service robot in the presence of a close observer will express a stronger need for relatedness (H1a), while those interacting with a stranger will place greater emphasis on popularity (H1b).

H1a Users express a higher need for relatedness when interacting with a service robot in presence of a close person (compared to an unknown person).

H1b Users express a higher need for popularity when interacting with a service robot in presence of an unknown person (compared to a close person).

### 3.2 Psychological user needs and expressivity

An interaction’s expressivity determines how noticeable and socially engaging a user’s interaction with a service robot is. When individuals prioritize relatedness, they are motivated to create shared experiences, which are best facilitated through higher expressivity to enhance social bonding. Similarly, when individuals seek popularity, they aim to attract attention and reinforce social visibility, making highly expressive interactions more desirable. Thus, we expect that a higher need for relatedness (H2a) and a higher need for popularity (H2b) will both be associated with a stronger preference for expressivity.

H2a A higher need for relatedness is associated with a higher preference for expressivity.

H2b A higher need for popularity is associated with a higher preference for expressivity.

### 3.3 Observer relationship, psychological user needs, and expressivity

Relatedness is prioritized in interactions with close others and is positively associated with expressivity, as noticeable interactions enhance shared experiences. Given that observer relationship influences relatedness needs, it follows that relatedness serves as a mediator between observer relationship and expressivity. Thus, we expect that the need for relatedness will mediate the effect of observer relationship on expressivity (H3).

H3 The need for relatedness mediates the effect of relationship to the observer on expressivity.

Building on prior arguments, popularity serves as a mediator between observer relationship and expressivity, but its effect is contingent on additional conditions. Since popularity is driven by social validation, individuals will prefer more expressive interactions only when they anticipate success and believe it reflects their own merit rather than external factors. Thus, success expectation and external attribution moderate the mediation of popularity, shaping whether individuals engage in noticeable interactions. Accordingly, we expect that popularity mediates the effect of observer relationship on expressivity (H4a), but this effect is moderated by success expectation (H4b).

H4 A moderated moderated mediation model adequately describes the relationship between observer, popularity, success expectation, external attribution, and expressivity:

o (a) Mediation: The need for popularity mediates the effect of relationship to the observer on expressivity.
o (b) Moderation: The effect of need for popularity on expressivity is moderated by success expectation.
o (c) Moderation: The moderation of the effect of need for popularity on expressivity by success expectation is in turn moderated by external attribution.

**FIGURE 1:**
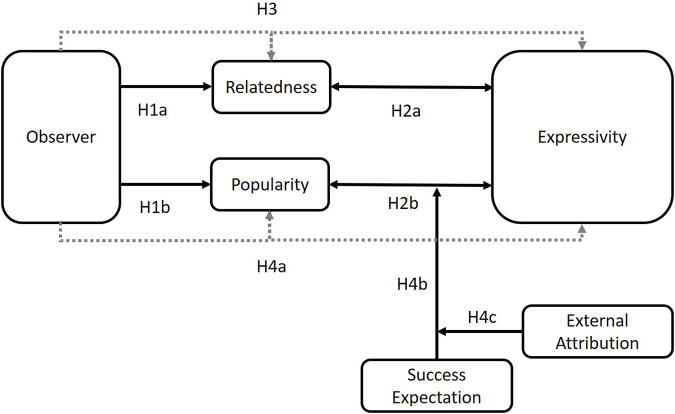
Summary model of study hypotheses.

The model of H4 as well as all other hypotheses are summarized in Figure 1.

## 4 Methods

### 4.1 Participants

The initial sample consisted of 367 German-speaking participants from Germany, Austria, and Switzerland, who were compensated €0,80 for approximately 5 min of participation (equivalent to the German minimum wage). The participants were recruited via Clickworker (clickworker.de), a crowdsourcing platform for scientific studies, and were informed about the compensation amount before the study. Following pre-registered exclusion criteria, participants who either failed the attention check (i.e., a question on the situation they ought to imagine), expressed trouble imagining the situation (i.e., a rating of less than four on a seven-point scale), or fell out of the admissible time to completion (i.e., below 180 or above 720 s) were excluded. This resulted in a final sample of 228 people. Of those, 58% were male, 42% female, and one person identified as non-binary. The average age was 40 years (M = 40.28; SD = 12.47; Med = 38), with the youngest participants being 18 and the oldest 73 years old.

### 4.2 Experimental design and Procedure

**FIGURE 2:**
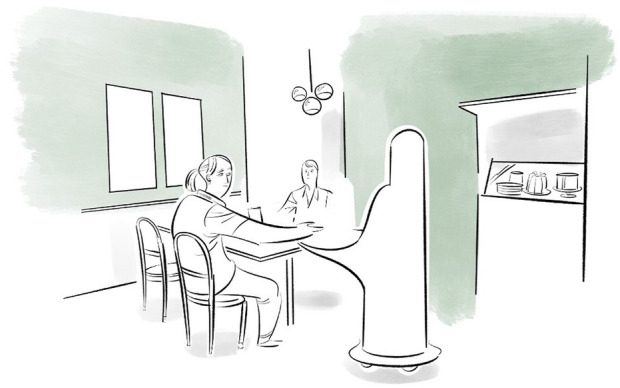
Schematic sketch of the described situation.

The experiment was conducted online as a between-subjects design with two conditions, close vs. unknown observer. Consequently, participants read one of two vignettes, describing the encounter with a talking service robot in presence of either a close or unknown person. This situation was additionally illustrated with a schematic sketch to support imagination and establish a common notion among participants (Figure 2). In general, the hospitality sector is a popular context in human-robot interaction research (Vishwakarma et al., 2023; Liu et al., 2024). We chose a café as it is a relatable public service setting that is not primarily associated with pragmatic concerns like privacy and performance (e.g., hospitals, offices) but foremost with experiential qualities like social exchange, leisure, and pleasurable goods (Hedaoo et al., 2019; Park et al., 2021).

After giving a consent agreement according to data protection laws, participants were asked to imagine one of the following two situations with a service robot in public.

#### 4.2.1 Close observer condition

You are in a café, where orders are taken by a talking robot. You are there in company, because a person close to you (e.g., a friend) is sitting at the table with you. S/he watches with interest as the robot approaches you, stops in front of you and asks, “May I take your order?”. Please take a moment to put yourself in the situation as you talk to the robot while being watched by the person close to you.

#### 4.2.2 Unknown observer condition

You are in a café, where orders are taken by a talking robot. You are there alone, but a person unknown to you is sitting at the opposite table. S/he watches with interest as the robot approaches you, stops in front of you and asks, “May I take your order?”. Please take a moment to put yourself in the situation as you talk to the robot while being watched by the stranger.

First, participants rated their ability to put themselves in this situation, and how much they expect a successful interaction with the robot. Subsequently, participants had to rate various statements according to how much they describe their ideal experience in that situation, i.e., the interaction experience they wish for. Then participants rated the situation according to who they would attribute an interaction failure to, themselves or the robot. Those central measures were the basis of the later analysis. Further exploratory items capturing additional needs, hedonic interaction qualities, comfort and social acceptability, impact of the observer, and alternative services, as well as attention check items are included in the openly available data set (see Data Availability Statement).

### 4.3 Key measures

All items were measured on a seven-point scale. Needs for relatedness and popularity in the imagined scenario were measured with four well-established items adapted from previous works (Sheldon et al., 2001; Hassenzahl et al., 2010). Expressivity, i.e., how noticeable participants would like their interaction to be, was also measured with four items (e.g., “Others shall experience how I interact with the robot”). Success expectation was assessed by means of three statements, for example, “I think the interaction with the robot will cause me no problems”. Participants’ tendency for external attribution was measured with three items (e.g., “It is not my fault if the order fails”). As no established scales existed for measuring those constructs in HRI, we created tailored items and preregistered our item analysis approach (see Data Availability Statement):

“Cronbach's α will be used to assess internal consistencies across all items pertaining to a construct. For psychological needs, if Cronbach's α is below .70, we will exclude items until that threshold is surpassed. For all other constructs, i.e., those with self-created scales, we will exclude items if their exclusion would lead to a higher Cronbach's α of the overall scale. If those prerequisites cannot be attained, we will still continue with our analysis based on the item aggregations that yield the largest internal consistency.”

No adaptations to the respective scales were necessary based on the data set. Table 1 shows an overview of these variables and corresponding measurement items. Descriptive statistics can be found in Table 2.

**TABLE 1:** Overview of key variables and corresponding items.


Relatedness (I want to have a sense of …)
… relatedness with people around me
… building a connection with those around me
… sharing a common experience with someone
… close intimacy with the people I am with
Popularity (I want to have a sense of …)
… being someone, others look to for guidance
… making a good impression on others
… being admired by others
… inspiring others with my behavior
Expressivity
Others shall experience how I interact with the robot
Others should be able to have a share in my interaction with the robot
I want others to notice what I do
I want others to witness how I interact with the robot
Success expectation
I think the interaction with the robot will cause me no problems
I am sure that I can handle the robot
I think I will succeed at ordering without any problems
External attribution
It is not my fault if the order fails
The robot is to blame, if the order goes wrong
It’s not up to me if the order does not work out

**TABLE 2:** Overview of internal scale consistencies and means (standard deviations).

Variable	Cronbach’s alpha	Overall	Close observer	Unknown observer
Relatedness	0.89	3.98 (1.49)	4.44 (1.42)	3.49 (1.40)
Popularity	0.86	3.74 (1.46)	3.75 (1.51)	3.73 (1.40)
Expressivity	0.92	3.29 (1.36)	3.45 (1.41)	3.11 (1.29)
Success Expectation	0.88	5.56 (1.03)	5.66 (0.99)	5.46 (1.06)
External Attribution	0.84	4.48 (1.23)	4.49 (1.27)	4.48 (1.20)

## 5 Results

### 5.1 Preliminary analyses

Internal scale consistencies (Cronbach’s alpha) as well as means and standard deviations for the measured variables, in each condition and overall, are displayed in Table 2. All scales met the pre-registered requirements and showed good to excellent internal consistency according to common conventions.

### 5.2 Group comparisons and correlations (H1 & H2)

First, we conducted an independent samples t-test to examine whether users in the close person condition express a higher need for relatedness (H1a). We found support for this assumption (*t* (226) = 5.09, *p* < 0.001, *d* = 0.67). Conversely, we expected them to express a lower need for popularity than people in the unknown person condition (H1b), which has not been the case (*t* (226) = 0.06, *p* = 0.95, *d* < 0.01). However, when conducting an exploratory within-subjects comparison with dependent sample t-test, people within the unknown person condition expressed a higher need for popularity than relatedness (*t* (109) = −2.63, *p* = 0.01, *d* = 0.25), while people within the close person condition expressed a higher need for relatedness than popularity (*t* (117) = 6.36, *p* < 0.001, *d* = 0.59). This indicates that while both groups did not differ in their need for popularity, people in the close person condition valued relatedness even higher than popularity, with the opposite being true for the unknown person condition.

Furthermore, we also found support for our second set of hypotheses that the needs for relatedness (H2a) and popularity (H2b) are associated with the preference for a more expressive interaction. The desired expressivity of the interaction significantly correlates with the need for relatedness (*r* (226) = 0.39, *p* < 0.001) as well as popularity (*r* (226) = 0.38, *p* < 0.001), see Table 3.

**TABLE 3:** Correlations between variables.

Variable	Relatedness	Popularity	Expressivity	Success expectation	External attribution
Relatedness	–				
Popularity	0.67*	–			
Expressivity	0.39*	0.38*	–		
Success Expectation	0.11	0.09	0.31*	–	
External Attribution	0.04	0.07	0.00	0.06	–

*p < .001.

### 5.3 Mediation and moderation analyses (H3 & H4)

**FIGURE 3:**
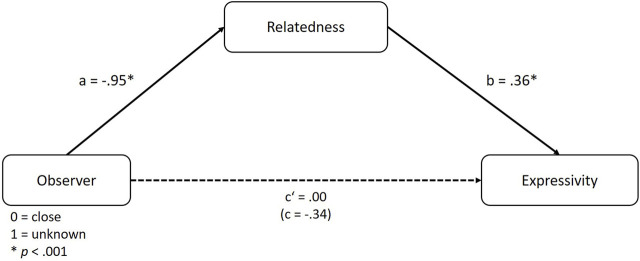
Mediation model according to H3.

Building on the former observations that the need for relatedness differed between conditions and is associated with higher expressivity, we continued by investigating our assumption that it serves as a mediator between the relationship to the observing person and expressivity preference. For this, we used the PROCESS macro, version 4.0, model 4 (Hayes, 2022) with bias-corrected 95% confidence intervals and 5,000 bootstrap samples. A graphical depiction of the resulting mediation model can be found in Figure 3.

The analysis showed no total effect of observer relationship on expressivity (*B* = −0.34, *SE* = 0.18, *t* = −1.89, *p* = 0.06), which also applies to the direct effect within the mediation model (*B* = 0.00, *SE* = 0.18, *t* = 0.02, *p* = 0.99). More importantly, analyzing the indirect effects, relatedness significantly mediates the effect of observer relationship on expressivity, supporting H3 (*B* = −0.34, *SE* = 0.10, 95% CI [-0.55, −0.18]). Dissecting the indirect path also corroborates the hypotheses H1a and H2a, since relatedness is significantly lower with unknown observers (*B* = −0.95, *SE* = 0.19, *t* = −5.09, *p* < 0.001) and relatedness positively affects expressivity (*B* = 0.36, *SE* = 0.06, *t* = 6.07, *p* < 0.001).

We also expected popularity to mediate between observer relationship and expressivity (H4a), but this mediation or the path between popularity and expressivity to be moderated by an interaction effect between success expectation (H4b) and external attribution of the outcome (H4c). In other words, we assumed that the user’s need for popularity is higher with an unknown observer. However, whether this popularity need also leads to a preference for expressivity is mainly dependent on, first, whether one expects to succeed in this situation and, second, whether one feels responsible for it (Figure 1). To this end, we applied the according model 18 in PROCESS (Hayes, 2022), again with bias-corrected 95% confidence intervals and 5,000 bootstrap samples.

Inevitably, this led to a rather complex model (for a comprehensive introduction to the concept of a moderated moderated mediation, see Hayes, 2018). Therefore, we report all results in Table 4 and concentrate here on the core results as well as a visual inspection of the relationships among the multiple variables. In sum, the model explains over 25% of the variance in expressivity (*R* ^2^ = 0.26, *MSE* = 1.43, *F* (8,219) = 9.51, *p* < 0.001). There was neither a significant direct effect (*B* = −0.28, *SE* = 0.16, *t* = −1.77, *p* = 0.08), nor an indirect mediation effect on any inspected level of the moderators. However, as expected, there is a significant three-way interaction between the mediator, i.e., need for popularity, with the moderators success expectation and external attribution, partially supporting H4.

**TABLE 4:** Results for the regression model (H4) with expressivity as criterion.

	B	SE	t	*p*
(Intercept)	7.36	−3.71	1.99	0.05
Observer^a^	−0.28	0.16	−1.77	0.08
Popularity (POP)	−1.71	0.93	−1.84	0.07
Success Expectation (SUC)	−0.73	0.64	−1.15	0.25
External Attribution (EXT)	−1.57	0.73	−2.14	0.03
POP * SUC	0.33	0.16	2.13	0.03
POP * EXT	0.47	0.18	2.54	0.01
SUC * EXT	0.25	0.12	1.99	0.05
POP * SUC * EXT	−0.08	0.03	−2.50	0.01

^a^ close = 0; unknown = 1

As can be seen in Table 4, there are also two-way interactions between the mediator and moderators as well as a main effect of external attribution. But given the significant higher order three-way interaction, those lower order effects have to be interpreted in context. To this end, a visual inspection of interaction plots at different levels of the predictors is pertinent to gauge the direction and magnitude of effects.

### 5.4 Visual inspection of moderated Moderated mediation effects (H4)

**FIGURE 4:**
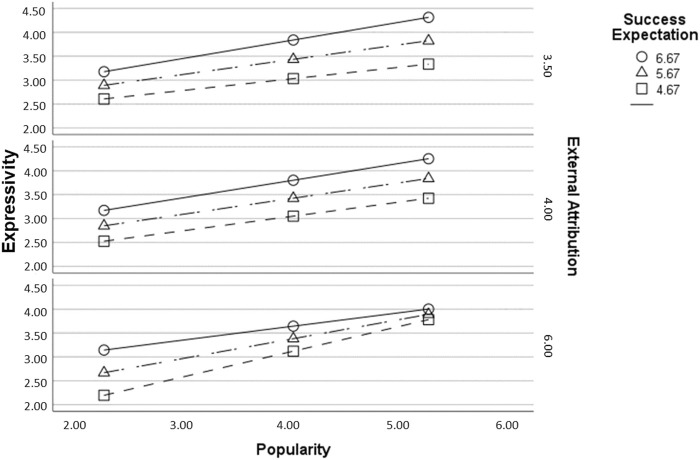
Graphical depiction of the moderated moderated mediation model of H4.

We used the code generated by PROCESS to visualize interactions and plotted three graphs with need for popularity on the x-axis and expressivity on the y-axis (Figure 4). Each of those graphs is based upon a different level of external attribution and contains three lines, each for one level of success expectation (PROCESS divides those moderators at the 16th, 50th, and 84th percentiles). This allows us to visually inspect tendencies that do not reveal themselves right away from the complex three-way interaction effect.

Figure 4 provides three observations that stand out and call for interpretation. First, all lines rise from left to right, indicating that, regardless of moderators, popularity is already positively related to expressivity, which is in line with H2b. Second, looking at the individual lines representing different degrees of success expectation, there tends to be a clear order with the circle line (high expectation) surmounting the triangle one (medium expectation), and the triangle line surmounting the square one (low expectation). This implies a higher preference for an expressive interaction at higher success expectations–in most ranges of popularity and at different degrees of external attribution. Third, however, this observation does not apply when there is a high need for popularity and external attribution is relatively high, as there is a near overlap of lines in the bottom graph at the right. Here, the lines seem to approach each other, indicating less of an influence of success expectation under these conditions. This aligns with the notion behind the whole interaction model: A higher need for popularity might lead to a higher preference for expressivity. However, if the outcome is externally attributed, success or failure probably have less influence on the user’s impression towards others, which attenuates the effect of success expectation on expressivity. In other words, those who do have lower success expectations have less reason to fear a suboptimal public interaction, and those with higher success expectations have a lower incentive to display their achievement. This is corroborated by the circle lines (displaying high success expectation), whose slope increases from the bottom to middle to top graph, i.e., with lower degrees of external attribution.

In sum, the assumed moderation of popularity need’s effect on expressivity by success expectation and external attribution is found within the data and supported by visual inspection.

## 6 Discussion

This research is motivated by the question of what the ideal interaction with service robots in public settings should look like. Our study shows that the expression of two different social needs varies with the relationship to the observer (close vs. unknown), which is in turn associated with the preference for the interaction’s expressivity. Depending on the respective need, however, this association may be moderated by certain preconceptions about the success and attribution of an interaction.

In line with our proposition, people express a higher need for relatedness when imagining an ideal interaction with a service robot in the presence of a (emotionally) close person compared to a stranger. We attribute this to the presence of another person being a necessary but not sufficient prerequisite for drawing pleasure from a shared experience. While people are motivated to share experiences with others, sharing itself does not automatically provide hedonic value (Jolly et al., 2019). As expected, the expressed need for relatedness also correlates with the preference for expressivity, i.e., an interaction that is noticeable and can be witnessed by others. Both observations point towards the conclusion that people are aware of the amplifying potential of co-experiencing a fairly new situation with a close other (Boothby et al., 2016) and therefore appreciate an interaction that involves more than themselves. In accordance with these observations, we could show that it is the psychological need for relatedness that mediates the effect between the relationship with the observer and the user’s wish to engage in an expressive interaction.

Regarding unknown observers, we assumed that the key source of a positive experience is the opportunity to present oneself favorably, leading to a higher need for popularity. Contrary to our proposition, participants did not express a higher need for popularity with an unknown observer compared to a close one. In other words, people were just as keen to present themselves favorably towards close others as they were towards strangers. However, when comparing the two needs within instead of between both experimental groups, results indicate that relatedness and popularity play different roles based on the relationship with the observer. When observed by strangers, people express a higher need for popularity than relatedness when asked about their ideal interaction. Moreover, we found support for our assumption that popularity as well is positively associated with the preference for an expressive interaction, as a perceivable interaction is a necessity to present oneself to others.

We anticipated that the connection between observer relationship and expressivity preference is not as simple when it comes to popularity compared to relatedness. While an interaction can fulfill one’s need for relatedness even if it does not go as preferred (Boothby et al., 2016), the need for popularity requires a successful interaction to experience achievement emotions from making a good impression on present others (Pekrun, 2000; Pekrun, 2006). Our findings support the assumption that the need for popularity resulting from being observed by a stranger mainly manifests in the preference for an expressive interaction if the user (1) tends to expect a successful interaction and (2) does not attribute this to external factors. This theoretical model accounted for more than 25% of the variance in expressivity, which seems like a moderately high share, given the fact that human behavior in social settings is subject to a myriad of potential factors.

In summary, the present study highlights the influence of the observer relationship on psychological needs and desired expressivity during service robot interactions. Understanding these dynamics can enhance the design and implementation of innovative technologies in public spaces.

### 6.1 Theoretical contributions

Our research contributes to the current literature in several regards. First of all, we investigated the effect of user-observer-relationship for a service experience from an ideal, positive perspective. Studies on how passive observers affect service experiences are still limited and they mostly consider instances of service failure (Fan et al., 2015; Weber et al., 2016; Qiu et al., 2018). On the contrary, we addressed a research gap by examining how those present others might enrich the service experience, which puts a new spin on this field of research and service robot encounters in particular. Notably, we investigated a situation that was inevitably non-private, i.e., people may have generally preferred an interaction with no present others. But as this is hardly attainable in public situations, our work can contribute to making the best out of these situations by understanding the psychological impacts of different observers on interaction preferences.

This also counterbalances the predominant pragmatic, aggregate-across-episodes approach to technology adoption represented by the TAM (Hornbæk and Hertzum, 2017). While its utilitarian focus on arguments like a product’s usefulness is indisputably valuable, our study adds to an increasing amount of current literature focus on hedonic determinants in service robot use (Lu et al., 2019; Alotaibi et al., 2022; Molinillo et al., 2022).

Moreover, the experiential approach applied by us instead of a pragmatic focus can serve as an example and inform further research, as it is better suited to some kinds of service environments. Usefulness has been shown to be a significant factor in people’s attitude toward service robot adoption in credence settings, e.g., hospitals, but not in service settings with an experience attribute, e.g., cafés (Park et al., 2021). As soon as service robots are able to provide the same quality of service a human would, the additional experiential value of interacting with an innovative technology might be a decisive factor in their adoption, especially if pragmatic considerations are not paramount.

By building on the premise that present others can also positively contribute to the experience of a service robot interaction, we added to strands of research that provide a counterpart to the often-applied social acceptability lens on public technology interactions (von Terzi et al., 2021). Socially acceptable design is to a large degree centered on reducing the negative effects that might come with public interactions, like disturbing others or looking awkward (Koelle et al., 2020). These concerns about how one’s technology use might affect others become even more relevant in service settings, like hotels, restaurants, or cafés, as they typically take place in public spaces (Qiu et al., 2018). Our study, however, emphasizes that the interaction expressivity, i.e., its capacity to be witnessed by others, is not inherently bad. On the contrary, while there are definitely concerns about one’s own impression toward others, as seen in the need for popularity, the fulfillment of those needs is a source of positive experiential value from a meaningful interaction (Hassenzahl et al., 2010). This contrasts the avoiding perspective implicitly dominant in social acceptability approaches and is in line with current insights on how not only failure avoidance but also positively framed achievement motivations can affect service robot adoption (Tojib et al., 2022).

Our study on service robot interaction also contributes to the larger field of user experience research. One pertinent theory of user experience builds on the proposition that a positive, meaningful interaction originates from the fulfillment of psychological needs that the respective context brings to the fore (Lenz et al., 2017; Hassenzahl et al., 2021). This fulfillment can emerge from the way an interaction is performed, i.e., if how it is done fits why it is done (Diefenbach et al., 2013). Our study is a well-fitting example of this approach and provides evidence for this theory. Regardless of whether people were more inclined to experience relatedness or popularity (i.e., the why), they also expressed a higher preference for expressivity (i.e., the how) when asked for an ideal interaction. This supports the notion that congruency between the reason for an interaction and the way it is performed creates positive experiences.

We also provided evidence that highlights the importance of theoretically differentiating the social needs an interaction responds to. Depending on the person present, people either prioritize need fulfillment from a shared experience or a favorable impression. While the former is straightforward, the latter calls for the consideration of circumstantial conditions. For popularity, in line with control-value-theory of achievement emotions (Pekrun, 2000; Pekrun, 2006), we could show that there are at least two circumstantial factors (i.e., success expectation and external attribution), which shape whether people, who wish to present them favorably, actually want their ideal interaction to be expressive. This not only supports control-value theory within a new application context but also highlights the relevance of context-sensitive design for creating positive user experiences.

### 6.2 Practical implications

The implications for context-sensitive design of service robots are one of the main contributions of our empirical exploration. Context sensitivity in this case is twofold: first, it demands awareness that the social environment affects user experiences from outside the typical interaction paradigm between user and technology. Second, it implies that there are factors within that social environment that people may react to differently. This in turn calls for customizable interactions, as service robots mostly operate in environments with a variety of potential users, observers, and thus user requirements, and it is still unclear how those interactions can be designed accordingly (Kong et al., 2018).

Service robots provide the sophisticated, specific skills needed to enable such customized interactions that reflect the customers’ needs and demands (Belanche et al., 2020b), but research is still focused on individual characteristics that may shape their general acceptance (Belanche et al., 2019). We aim to shed light on the importance of considering the social context in the design of public interactions and the potential to create more engaging, satisfying, and meaningful experiences–even if the people around us are strangers. In response, our study promotes expressivity as a key design factor that characterizes public interaction with a robot.

This provides implications for how robots in service settings should be designed. The service robot in our café scenario may react to whether the customer is sitting alone or in company and may adapt the expressivity of an interaction accordingly. For example, Pepper, a popular robot for social purposes (Pandey and Gelin, 2018), usually speaks with its users but may also communicate through texts on a display mounted on its chest. These modalities are inherently different in how expressive and therefore noticeable corresponding interactions are, which allows adjusting expressivity to whether the customer is alone or in company. Interventions with expressivity in mind could also be more fine-grained. Font size and graphics of a terminal could be enhanced to be visible from afar. Displays could be curved to be visible to people not directly in front of it. Or the volume of voice interfaces could be regulated situationally. As soon as it is clear that people may embrace the fact that their interaction is noticeable, this results in a range of conceivable design implications.

The possibility to adjust the expressivity of an interaction (or at least the opportunity to opt out of a spoken dialogue) seems especially important as we identified not only relatedness with known others but also popularity in front of unknown observers as a source of positive experiences. Even if one may enjoy a service alone, they might still be inclined to let others witness their interaction. A potential pitfall in this situation, however, can be the possibility of something going wrong while ordering and thus embarrassing the user, which is reflected in the moderating effects of success expectation and its external attribution. But within this insight, there also lies a solution through considerate interaction design. In the pilot phase of introducing service robots, their adoption could be fostered by letting the robot take accountability. We suppose that people will be less concerned about the expressivity of their interaction if the service robot approaches them by explaining that they are still in the early stages of their training and therefore apologize in advance for any inconvenience. This can take pressure off the user by fostering external attribution (also for the people within earshot) and may even enhance the feeling of personal achievement if everything goes as expected. All in all, the study results stress the importance of designing service robots (interactions) that facilitate success and positive attribution, especially when interacting in front of strangers.

### 6.3 Future research and limitations

Limitations of the current study and resulting further research questions refer to two broader aspects. The first aspect concerns the study’s design. We conducted a vignette study to explore the fundamental notion of the positive potential of expressivity in a public service robot interaction. Even though vignette studies are a valid source of systematically investigating effects in a controlled manner (Aguinis and Bradley, 2014), the hypothetical nature of our survey calls for more realistic follow-up studies where people actually encounter the pros and cons of interacting in a public setting. This would mean that expressivity could also be experienced instead of having potential users indicating their desired degree of it. Since we provided a low-detailed sketch and description of the robot in our study to facilitate participants’ ability to put themselves in the described scenario, we also somehow limited the room for imagination regarding its expressivity. Allowing an actual, broad-range variation of this expressivity in future experiments would provide an opportunity to further scrutinize our conclusions regarding the design of expressive features of the human-robot-interaction.

Furthermore, we asked for psychological need fulfillment in an ideal interaction with rating scales, which allowed people to rate relatedness and popularity independently. However, this may not fully account for practical limitations in the design of interactions, as the fulfillment of one need may inhibit that of another (Hassenzahl et al., 2010). While evidence supported our assumptions regarding the paramount role of each one of those needs under different observer conditions, our approach of analyzing them independently does not take into account the interaction effects that could arise when both needs are simultaneously active. Similarly, the topic of incommensurability may also apply to user and observer needs. Future research should additionally measure the observer’s needs in the respective situation, since the fulfillment of a user’s need for relatedness, for example, may inhibit an observer’s need for autonomy.

The second aspect relates to the generalizability of results. There are several ways our applied scenario may vary in a real-life setting and those variations have to be further examined. Although we justified our focus on a café as an experiential setting rather than a credence setting like a hospital, it remains unclear whether the interaction is more outcome-focused (e.g., receiving treatment or service) rather than process-oriented (e.g., having a pleasant experience). Gonzalez-Aguirre et al. (2021) provide a review of the uses and applications of service robots across various operational areas, offering further inspiration for alternative settings. We also did not consider individual, intrapersonal factors in our study. Previous research has shown that, e.g., the emotional state of a user, influences how satisfied they are with the service of a robot (Lajante et al., 2023). Therefore, future studies could investigate the influence of user factors on the associations we found.

Furthermore, audiences may vary and we do not know yet how this affects the optimal user experience. We applied a stripped-down design with a single observer (whose presence can already have decisive effects; Guerin and Innes, 1984) and manipulated the user-observer relationship. However, users may be accompanied by someone they barely know, or by several people, or find themselves in a crowded environment, all of which influence the ratio of close to unknown observers. People may also differ, for example, in their age, gender, appearance, or cultural background, and may therefore behave differently in the examined service setting (Fan et al., 2015). It seems promising here to focus less on the sheer endless number of possible combinations but on the strength of emerging psychological needs. For example, applying social impact theory (Latané, 1981), future research could focus on how the need for relatedness and popularity is a function of the strength (i.e., importance), number (i.e., how many persons), and immediacy (i.e., proximity) of the social source or the potential audience (Qiu et al., 2018).

Similarly, our study did not account for the potential absence of any observer as a reference scenario. Our study focused on service robot interactions specifically in public settings where the presence of at least one observer is highly probable. While prior HRI research has primarily focused on user–robot dyads in a vacuum, we aimed to extend these insights by examining how even a single observer affects user motivations and preferences in inevitably non-private interactions. We would anticipate that, in the absence of any observers, users would place less emphasis on being noticed (popularity) or sharing experiences (relatedness). Future research could include such a baseline to more definitively quantify the influence of any observer’s presence.

## 7 Conclusion

The current research explored the ideal interaction with a service robot in a public setting from a performative perspective. It provided support for the notion that an expressive, thus noticeable interaction is not necessarily unpleasant but may allow people to fulfill their basic psychological needs of relatedness or popularity, depending on who witnesses their interaction. At the same time, we discovered potential pitfalls and design implications that must be addressed when people seek to draw pleasure from presenting themselves favorably when interacting with a public technology. While our focus was on service robots, these insights on HCI in a public setting may also encourage fellow researchers and designers to explore expressivity in the interaction with other innovative technologies, considering the presence of others less as a constraint and more as a resource of positive user experiences.

## Data Availability

The datasets presented in this study can be found in online repositories. The names of the repository/repositories and accession number(s) can be found below: https://osf.io/v54qk/.
